# Causes and risk factors for common mental illnesses: the beliefs of paediatric hospital staff in the United Arab Emirates

**DOI:** 10.1186/s13033-020-00367-6

**Published:** 2020-05-24

**Authors:** Shameran Slewa-Younan, Thomas P. Nguyen, Nabeel Al-Yateem, Rachel Cathrine Rossiter, Walter Robb

**Affiliations:** 1 Mental Health, Translational Health Research Institute, School of Medicine, Humanitarian and Development Research Initiative, Western Sydney University, Penrith South DC, Sydney, NSW 1797 Australia; 2 Centre for Mental Health, Melbourne School of Population and Global Health, University of Melbourne, Melbourne, Australia; 3 Mental Health, School of Medicine, Western Sydney University, Sydney, Australia; 4 Department of Nursing, College of Health Sciences, University of Sharjah, Sharjah, United Arab Emirates; 5 School of Nursing, Midwifery and Indigenous Health, Faculty of Science, Charles Sturt University, Orange, NSW Australia; 6 Research Institute for Medical and Health Sciences (RIMHS), University of Sharjah, Sharjah, United Arab Emirates; 7 School of Nursing, Midwifery & Indigenous Health, Charles Sturt University, Orange, NSW Australia; 8 University of Salford, Greater Manchester, UK; 9 Quantitative Analysis, Analyse Australia, Brisbane, Australia

**Keywords:** Mental health literacy, Professional health education gaps, Paediatric care, Cross-cultural research

## Abstract

**Background:**

Children and adolescents with chronic physical health conditions are vulnerable to poor mental health outcomes. The measurement of mental health literacy of health professionals working with such populations is important because of their role in promoting early and appropriate help-seeking. This study sought to determine the beliefs regarding the causes of and risks factors for three types of mental illnesses amongst health professionals in United Arab Emirates.

**Method:**

A culturally validated mental health literacy survey presenting three vignettes of fictional characters meeting diagnostic criteria for posttraumatic stress disorder, depression with suicidal thoughts and psychosis was distributed. The survey measured health care professionals’ beliefs regarding the causes of and risk factors for these disorders.

**Results:**

A total of 317 health care professional (> 90% nurses) were surveyed from across the UAE. Although 43.8% correctly endorsed exposure to a ‘traumatic event’ as the most likely cause for developing posttraumatic stress disorder, there was a more limited understanding of the contribution of biopsychosocial factors to the development of the mental illness, particularly for psychosis. Participant socio-demographic variables were associated with attributions of religious or spiritual beliefs and personal weakness as causal and/or vulnerability factors in the development of depression with suicidal thoughts and psychosis.

**Conclusions:**

Efforts to improve mental health systems and health care providers in UAE and other similar Middle Eastern countries requires targeted mental health literacy programs that seek to integrate biopsychosocial models of mental illness and their treatment with the positive aspects of religious and cultural beliefs that are dominant in this region.

## Background

The United Arab Emirates (UAE) is located in Western Asia, situated at the tip of the Arabian Peninsula, sharing maritime borders with Qatar and Iran and land borders with Saudi Arabia and Oman [1]. It was established in 1971 as a federation of seven emirates and is well regarded in its region due to its solid economy, tax free status and high standard of living [1]. The UAE has a very large expatriate demographic, with foreigners making up 84% of the population [2]. Despite the rapid growth in wealth and the high standard of living of the UAE, rates of chronic health conditions in children remain problematic and this is particularly relevant in a country where approximately 35% of its total population are aged between 0 and 24 years [3].

Children and adolescents with chronic physical health conditions face multiple challenges such as prolonged periods of poor health and repeated hospitalisations [3].This often leads to impairments in educational and social functioning and can be associated with poor mental health outcomes such as anxiety and depressive conditions. If left undiagnosed and untreated, these psychiatric conditions can continue into adulthood, leading to long-term reductions in quality of life [4].

Eapen et al. found that that more than two-fifths of Emirati children presenting to a primary care provider in Al Ain had a diagnosed mental illness [5]. However, only 1.1% of the sample presented to their general practitioner primarily for their psychiatric symptoms. Most concerningly, in a separate study, only 38% of parents in a sample of UAE citizens stated that they would seek specialist mental health care if their children or family member was developing a psychiatric problem [6]. In the Arab culture, having a mental illness remains stigmatised and help-seeking behaviours are often viewed as bringing shame on the family and the affected person may be attributed to a weakness in one’s faith [7]. This reluctance to seek professional help is exacerbated by the acceptability of non-professional traditional sources of help such as religious advice as the first option for support. The shortage of qualified mental health clinicians further influences decisions to seek medical support [7].

Help-seeking behaviour in the Emirati paediatric population is largely shaped and arguably hindered by parental perceptions and attitudes [6]. More importantly, children with psychological problems will often present with somatic and physical symptoms as the expression of emotional distress is not common in the Arab culture [6, 7]. Thus, there is a strong imperative for health professionals in the UAE to demonstrate adequate *mental health literacy* to enable them to identify and support children with comorbid chronic health conditions and mental illness, in a context characterised by the interaction of complex cultural nuances.

Mental health literacy (MHL) refers to the, “knowledge and beliefs about mental disorders which aid their recognition, management or prevention” [8]. Research has shown that increased knowledge about mental illness and the understanding of where to seek help and treatment can promote early identification, increase the use of mental health services and improve mental health outcomes [9]. Al-Yateem et al. previously noted that problem recognition and the ability to identify appropriate, evidence-based interventions was limited in a sample of paediatric hospital staff in the UAE [3]. For example, when presented with a vignette describing posttraumatic stress disorder (PTSD), only 47% of the sample correctly identified the problem described and only 54.3% were able to recognise psychosis [3].

Another potentially important aspect of MHL is the impact of the clinicians’ knowledge and beliefs concerning risk factors and causes of mental illnesses. Evidence suggests that knowledge of causes of mental illness and cultural beliefs may play a role in help-seeking behaviour [10], although there is limited knowledge in this regard particularly within the UAE context. The Western understanding of risk factors for and causes of mental illness are encompassed within the biopsychosocial model. This model views the causation of disease as a complex interaction between biological (e.g. genetic, biochemical), psychological (e.g. developmental experiences, personality style) and social (e.g. family and cultural) factors, with treatment approaches often addressing these dynamic influences [11].

Whilst this model underpins the current approach to treating mental illness in Westernised countries, there is a wider acceptance of the practice in the Arab world of seeking advice from religious faith healers (Mattawa) before asking for professional help. The Mattawa may not necessarily use medical or psychological frameworks for treatment but may instead recite prayers from the Qur’an or elicit traditional medicine to drive the ‘evil spirits’ or the ‘evil eye’ away [7]. This stems from a belief that one of the causes of mental illness may be possession by an unhelpful jinn (spirit being) taking over one’s life [12]. Recognising this, researchers are increasingly calling for mental health systems to consider the impact of religious beliefs on the management of mental illness and to consider how spirituality and religion can be incorporated into psychotherapeutic practices in order to ensure treatment is acceptable and effective [13].

Given the cultural diversity of the UAE’s expatriate population, developing an understanding of paediatric health staff knowledge of the causes of and risk factors for developing mental illness is important. Such information is vital to inform the development of culturally sensitive health promotion training to support these healthcare professionals in recognising and appropriately dealing with children who may be showing signs of mental illness.

With these considerations in mind and noting the paucity of the relevant literature, no specific directional hypotheses were postulated. Instead, we were interested in developing an evidence base on the MHL of paediatric hospital staff with regards to their beliefs concerning the causes of and the risks factors associated with three mental illness, namely, PTSD, depression with suicidal thoughts and psychosis. However, categorisation using the biopsychosocial model approach was used to frame the causal factors in order to highlight the domains more strongly endorsed by participants.

## Methods

### Study design and participant recruitment

A cross-sectional approach utilising participants recruited via convenience sampling of the total accessible population was undertaken. Of the 104 hospitals in the UAE, a total of 71 private hospitals were excluded from the final sample as access to their staff was refused. The remaining 33 government hospitals were sites either managed by the government or by independent authorities such as the Health Authority of Abu Dhabi, and the Dubai Health Authority. Of these hospitals, the main providers of paediatric care in each emirate (n = 13) were identified and contacted to access potential participants. Ethics approval to conduct this study was gained from seven hospitals, representing the following six emirates: Sharjah (n = 1); Dubai (n = 2); Abu Dhabi (n = 1); Ajman (n = 1); Ras Al-Khaimah (n = 1); and Umm Al-Quwain (n = 1). The remaining emirate (Fujairah) makes up a small proportion of the total UAE population.

Nurses and doctors who provide care for children from these seven hospitals were invited to participate in the study. Each hospital had a liaison person who would distribute and collect either an online or paper-based questionnaire, according to the preference of the participating hospitals. As this process was centrally administered, we could not accurately determine the number of staff members who were given a copy of the questionnaire. However, we estimate that there were a maximum of 1400 doctors and nurses in total that are employed in the participating paediatric hospital units. In total, 379 healthcare professionals responded to the survey, giving a minimum response rate of 27% (if all 1400 staff received either a hard copy or online questionnaire.) Of these 379 responses, 324 had responded to at least one of the clinical MHL vignettes. Although response rates were not consistent across all hospitals, the majority of responses came from the largest hospitals. Moreover, nurses made up a significantly greater proportion of responses in comparison to medical staff. However, the final sample is representative of the broad socioeconomic demographic of the UAE population and healthcare system, sans private hospitals.

### Measures

#### MHL survey

The survey used was modelled off the MHL survey reported by Jorm et al. [8] and later adapted in Slewa-Younan et al. [14], following permission from those authors. The vignettes in the MHL questionnaire used were further modified for this study by authors NA and RR, following consensus that the vignettes would be culturally valid and reflective of diagnostic criteria for the three disorders as stipulated in the Diagnostic and Statistical Manual of Mental Disorders (DSM) Fifth Edition [15]. More specifically, the names in the vignettes were changed to Arabic names (i.e., Miriam, Abdul, and Saed) and certain phrases were amended (e.g., “Reading Bible” was changed to “Reading Koran or Bible” and “Talking with priest” to “Talking with religious person or priest”). The survey was kept in English despite Arabic being the official language of the UAE as English is commonly spoken by all healthcare professionals in the Emirati health system. Using Arabic would have excluded a large number of expatriate healthcare professionals who would not have been able to complete the survey otherwise. The questionnaire was then piloted with 12 final-year Bachelor of Health Science (Nursing) students to determine cultural acceptability. These students were representative of the cultural and linguistic diversity of the study’s target population study (i.e., Arabic, Indian, and Filipino). Following this pilot testing, no further changes were required.

The three case vignettes used in the questionnaire were all fictional characters. The first (Miriam) suffered from PTSD, the second (Abdul) experienced depression with suicidal thoughts, and the third (Saed) displayed psychotic symptomatology. Following each vignette, participants were firstly asked, ‘How likely do you think each of the following is to be a factor in this sort of problem developing in anybody?’ Participants were presented with a number of possible causes that included biological, social or psychological causes e.g. ‘genetic or inherited’, ‘coming from a worn torn country’ or ‘family problems’. From these causes, participants were asked to rate each of the mentioned items as ‘very likely’, ‘likely’, ‘not likely’ or ‘depends/don’t know’. They were then asked to select the option that they believed was ‘most likely’ to be the cause for the condition. Participants were also asked about the risk factors for developing a problem like the ones described in each of the vignettes. They were asked ‘Do you think each of the following people would be ‘very likely’, ‘likely’, ‘not likely’ or ‘depends/don’t know’ to develop a problem like (Miriam/Abdul/Saed)’s?’ They were then asked to select the option that they believed was ‘most likely’ to increase vulnerability (risk factor) for the conditions. Sixteen options were presented such as ‘those unemployed’, ‘women’ and ‘people who are single/or on their own’. It should be noted that when participants were considering possible causes and risk factors for the psychosis vignette (Saed) an additional option of ‘use of forbidden drugs’ was provided, (this was not an option for the two other vignettes).

### Statistical analyses

Responses to causes and risks were recoded into ‘likely’ (this collapsed ‘very likely’ and ‘likely), ‘not likely’, ‘undecided’ (this include ‘depends’ and ‘don’t know’) and missing. Statistical analyses were used to compare the responses between vignettes. Specifically, a test of proportions was used to compare the percentage of respondents selecting ‘likely’ for within each of the causes and risks. All significant p-values cited in these comparisons of proportions are after Bonferroni correction was applied. The influence of each of the socio-demographic factors (gender, age group, region of origin, years in UAE, language, qualifications, and years of experience) on each of the causal and risk factors selected as ‘likely’ by participants was examined by logistic regression to gauge their effect in the presence of all other socio-demographics. Analyses were performed using the freely available R software version R x64 3.5.2 [16].

#### Missing values

Missing responses to the likelihood of each cause or risk are shown as percentages in Tables 2 and 3. Where there were missing responses to the ‘most likely’ (one choice only) cause or risk, percentages were calculated from respondents only. The numbers of respondents are shown in the tables. As noted previously, in comparing the percentage of ‘likely’ causes and risks associated with the vignettes, the response category for the psychosis vignette “People who use drugs” was excluded because it did not apply to the other vignettes.

### Ethics approval and consent to participate

Ethics approval was obtained from the University of Sharjah Research Ethics Committee (REC, ref# REC-23-11-15-46) and the research ethics committees of health services in the areas from which participants were drawn (DHA-ref# DSREC-12/2015-13; MOH-ref# R04). Return of the questionnaire was considered as evidence that respondents had consented to participate in the study.

## Results

A total of 379 healthcare professionals responded to the survey. From this total, 317 completed the PTSD vignette, 281 completed depression with suicidal thoughts vignette and 208 responded to the psychosis vignette. The majority of participants were nurses (92.9%); the remainder was medical doctors (7.1%). Most participants were females (90.7%) and aged 30–39 years (43.4%). A large proportion of participants were from the Indian sub-continent; followed by the Middle East, UAE nationals, South East Asia, and Africa; over one-third (38.3%) did not report their nationality. The main spoken languages were Arabic (37.2%) or an Indian language (35.9%), and 15.4% reported English as their mother tongue. A majority (58.5%) of nurse participants had a diploma-level qualification and 59.1% of participating medical doctors had at least a bachelor’s degree. Finally, 83.1% of all participants had at least 5 years of experience, and 53.7% worked in outpatient departments (OPDs).

Participants’ demographic characteristics according to completed MHL vignettes are presented in Table 1.

**Table 1:** Demographic characteristics of the study participants collapsed according the MHL surveys completed

All participants	PTSD		Depression with suicidal thoughts		Psychosis	
	n	%	n	%	n	%
	317	100	281	100	208	100
Gender						
Female	274	86.4	243	86.5	181	87.0
Male	33	10.4	33	11.7	25	12.0
Missing	10	3.2	5	1.8	2	1.0
Age group						
20–29	79	24.9	64	22.8	45	21.6
30–39	129	40.7	122	43.4	98	47.1
40–49	74	23.3	69	24.6	47	22.6
50–59	18	5.7	17	6.0	13	6.3
60+	2	0.6	2	0.7	2	1.0
Missing	15	4.7	7	2.5	3	1.4
Region						
Middle east	40	12.6	37	13.2	25	12.0
A frica	13	4.1	13	4.6	9	4.3
Sub-continent	119	37.5	117	41.6	98	47.1
South East Asia	23	7.3	22	7.8	19	9.1
UAE	32	10.1	23	8.2	12	5.8
Missing	90	28.4	69	24.6	45	21.6
Years of residency in UAE						
9 or less	81	25.6	80	28.5	71	34.1
10–19	53	16.7	51	18.1	39	18.8
20–29	40	12.6	35	12.5	23	11.1
30+	40	12.6	38	13.5	30	14.4
Missing	103	32.5	77	27.4	45	21.6
Language						
Arabic	84	26.5	72	25.6	47	22.6
English	35	11.0	34	12.1	29	13.9
India	84	26.5	81	28.8	67	32.2
Philippines	20	6.3	19	6.8	16	7.7
Other	6	1.9	7	2.5	0	0.0
Missing	88	27.8	68	24.2	49	23.6
Profession						
Medicine	27	8.5	14	5.0	6	2.9
Nursing	290	91.5	267	95.0	202	97.1
Qualification						
Nursing Diploma	116	36.6	114	40.6	85	40.9
Nursing B.Sc.	75	23.7	73	26.0	65	31.3
Nursing post-graduate qualification	11	3.5	11	4	8	3.9
Medical B.Sc.	13	4.1	3	1.1	2	1.0
Medical post-graduate qualification	9	2.9	8	2.8	2	1.0
Other	1	0.3	1	0.4	0	0.0
Missing	92	29.0	71	25.3	46	22.1
Years of experience						
0– < 5	53	16.7	47	16.7		
5– < 10	81	25.6	70	24.9	55	26.4
10– < 15	48	15.1	45	16.0	35	16.8
15– < 20	51	16.1	46	16.4	31	14.9
20+	68	21.5	65	23.1	46	22.1
Missing	16	5.0	8	2.8	4	1.9
Work setting						
Emergency Department	17	5.4	17	6.0	14	6.7
Outpatient Department	164	51.7	152	54.1	125	60.1
Pediatric wards	112	35.3	91	32.4	52	25.0
Pediatric Intensive care unit	10	3.2	11	3.9	9	4.3
Missing	14	4.4	10	3.6	8	3.8

### Causes beliefs

Table 2 presents the rates of endorsement for causal factors for each of the clinical vignettes.

**Table 2:** Rates of endorsement of causal factors by clinical vignette

Causal factors	PTSD vignette (n = 317)					Depression with suicidal thoughts vignette (n = 281)					Psychosis vignette (n = 208)				
	Likely (%)	Not likely (%)	Undecided, depends or don’t know (%)	Missing (%)	Most likely (one choice allowed)^a^ (%) n = 297	Likely (%)	Not likely (%)	Undecided, depends or don’t know (%)	Missing (%)	Most likely (one choice allowed)^a^ (%) n = 281	Likely (%)	Not likely (%)	Undecided, depends or don’t know (%)	Missing (%)	Most likely (one choice allowed)^a^ (%) n = 208
Biological domain															
Having a parent or parents with psychological problems	63.4	19.9	13.6	3.2	5.4	77.9	10.7	9.3	2.1	11.4	78.8	10.1	9.6	1.4	13.3
Poor physical health	41.3	32.8	19.6	6.3	0.3	47.3	24.6	21.7	6.4	1.8	39.4	34.1	20.7	5.8	2.1
Genetic or inherited	34.7	42.3	17.7	5.4	2.0	49.1	30.6	16.7	3.6	5.9	58.7	23.6	15.4	2.4	14.4
Psychological domain															
Having a bad childhood	72.9	14.8	9.5	2.8	10.1	83.6	7.5	6.0	2.8	18.0	76.9	13.5	9.1	0.5	12.2
Being a person with a weak character	57.4	16.1	22.7	3.8	4.4	72.2	12.1	13.9	1.8	15.1	64.4	13.0	20.7	1.9	15.4
Social domain															
Experiencing a traumatic event	87.4	3.8	6.0	2.8	43.8	76.2	12.1	8.9	2.8	18.0	78.4	9.1	11.1	1.4	18.6
Coming from a war-torn country	78.2	9.1	9.5	2.8	20.9	65.1	19.9	11.0	3.9	3.3	70.2	15.9	11.5	2.4	2.7
Moving to a new country	70.7	11.4	13.6	4.4	7.1	63.0	13.2	19.6	4.3	2.9	49.5	22.1	26.9	1.4	0.0
Family problems	69.1	16.7	9.8	4.4	5.1	82.6	7.1	7.8	2.5	21.7	80.8	8.2	10.1	1.0	18.1
External higher power domain															
Punishment from God	13.9	61.2	20.2	4.7	0.0	11.7	58.0	26.0	4.3	0.0	12.0	61.1	25.0	1.9	0.0
Evil eye	12.9	60.6	21.8	4.7	0.3	14.6	55.9	23.8	5.7	0.0	15.4	61.5	20.7	2.4	0.5
The problem is destiny	30.0	40.1	23.0	6.9	0.7	26.3	44.5	23.5	5.7	1.8	26.4	44.2	23.6	5.8	2.7

^a^These percentages add to 100%

When participants were asked to consider the clinical vignette of PTSD, the top three causal factors endorsed as ‘likely’ were ‘experiencing a traumatic event’ ‘coming from a war torn country’ and ‘having a bad childhood’. In regards to depression with suicidal thoughts, the top three ‘likely’ factors were ‘having a bad childhood’, ‘family problems’ and ‘having a parent or parents with psychological problems’. For the psychosis vignette ‘family problems’, ‘having a parent or parents with psychological problems’ and ‘experiencing a traumatic event’ were the top three causal factors endorsed as ‘likely’ to cause that type of mental illness. Interestingly, there were some consistency and differences were noted when participants were asked to nominate the ‘most likely’ (only one choice allowed) cause for the each of the clinical vignettes. As previously noted, ‘experiencing a traumatic event’ was selected by 43.8% of participants as the top cause for the PTSD vignette indicating a strength of endorsement in this factor. In contrast, the lead cause for depression with suicidal thoughts ‘family problems’ was selected by 21.7% of participants. Of note was ‘experiencing a traumatic event’ selected by 18.6% as the top cause for psychosis.

### Beliefs regarding vulnerability for developing PTSD, depression with suicidal thoughts and psychosis

Table 3 presents the rates of endorsement for the risk factors for development of each of the clinical vignettes.

**Table 3:** Rates of endorsement of risk factors by clinical vignette

Risk factors	PTSD vignette (n = 297)					Depression with suicidal thoughts vignette (n = 272)					Psychosis vignette (n = 188)				
	Likely (%)	Not likely (%)	Undecided depends or don’t know (%)	Missing (%)	Most likely (one choice allowed^a^ (%) n = 295	Likely (%)	Not likely (%)	Undecided depends or don’t know (%)	Missing (%)	Most likely (one choice allowed)^a^ (%) n = 260	Likely (%)	Not likely (%)	Undecided depends or don’t know (%)	Missing (%)	Most likely (one choice allowed)^a^ (%) n = 146
Born in a war-torn country	72.2	8.2	16.1	3.5	60.0	68.7	10.0	17.4	3.9	30.0	61.5	14.4	21.6	2.4	19.9
Unemployed	58.7	18.9	19.2	3.2	9.2	67.6	11.0	17.1	4.3	25.0	66.3	10.1	21.2	2.4	39.7
People who are single/or on their own	49.2	24.6	22.7	3.5	7.5	51.6	19.6	23.8	5.0	10.8	48.1	18.8	30.8	2.4	9.6
Women	45.4	24.0	27.1	3.5	3.4	39.1	29.2	27.8	3.9	1.9	30.8	36.1	30.8	2.4	1.4
Served in the army	42.9	22.7	30.9	3.5	4.1	43.1	24.2	27.4	5.3	2.7	33.2	33.2	31.3	2.4	4.8
People who are poor	36.6	28.1	30.0	5.4	1.7	45.6	23.1	27.4	3.9	5.0	37.5	29.8	29.3	3.4	0.7
Older people	35.3	30.9	30.0	3.8	1.7	38.1	23.8	33.5	4.6	1.9	25.0	36.5	35.6	2.9	5.5
Young people	30.9	31.5	31.9	5.7	3.1	40.2	25.6	31.0	3.2	2.7	35.1	31.7	31.3	1.9	2.1
Employed	30.3	38.5	26.8	4.4	3.7	40.9	31.7	23.1	4.3	7.7	30.3	43.8	24.0	1.9	10.3
Men	29.0	30.0	36.6	4.4	0.3	34.9	29.9	31.3	3.9	1.9	31.7	30.3	35.6	2.4	2.1
People who have families	28.1	39.7	28.7	3.5	1.7	33.8	38.8	23.1	4.3	3.1	21.2	41.8	35.1	1.9	0.0
Those who are not very religious	28.1	29.7	36.3	6.0	2.0	29.5	30.2	32.7	7.5	3.5	22.1	35.6	38.5	3.8	1.4
People who are rich	24.9	33.1	36.6	5.4	0.0	26.0	35.2	32.7	6.0	0.4	20.7	37.5	38.9	2.9	0.0
Those who are very religious	14.5	49.8	31.2	4.4	0.7	18.1	41.6	35.6	4.6	1.9	18.8	41.8	35.1	4.3	2.1
Being from a Christian background	10.4	50.8	34.4	4.4	0.0	11.4	52.7	32.0	3.9	1.2	7.7	55.3	35.1	1.9	0.7
Being from a Muslim background	8.2	55.2	32.5	4.1	1.0	8.5	54.8	29.9	6.8	0.4	13.5	51.0	32.2	3.4	0.0

^a^These percentages add to 100%

Participants selected ‘coming from a war-torn country’, being ‘unemployed’ and ‘people who are single or on their own’ as the top three ‘likely’ risk factors to increase vulnerability to develop all three disorders of PTSD, depression with suicidal thoughts and psychosis. However, when asked to select only one risk factor some differences between vignettes emerged. For both the PTSD and depression with suicidal thoughts vignettes ‘born in war torn country’ was by far the top risk factor selected by 60% and 30% of all participants respectively. However, for the psychosis vignette, being ‘unemployed’ was considered by 39.7% of participants as the ‘most likely’ factor to increasing vulnerability for this type of mental illness.

### Associations between socio-demographic characteristics on the selection of causes and risk factors to the three vignettes

The logistic regression models found a small number of instances where a socio-demographic variable was significantly associated with the respondents’ selection of likely cause or risk, when considered in the context of all other socio-demographic variables.

#### PTSD Vignette

Respondents with a nursing background were more likely to endorse ‘poor physical health’ as a causal factor for the PTSD vignette than those from medical background (p = 0.033). However, those with more experience were more likely to select ‘having a parent or parents with psychological problems’ as cause for PTSD (p = 0.049). With respect to risk factors, females were more likely to select ‘those who are not very religious’ as increasing vulnerability to PTSD (p = 0.028). Those who spoke Filipino compared with Arabic were more likely to select ‘people with families’ as a risk factor for development of PTSD (p = 0.046).

#### Depression with suicidal thoughts

There was a significant increase in the selection of the causal factor ‘the problem is destiny’ for the depression vignette as respondents increased in age (p = 0.041). Those respondents who had resided in UAE for 10 years or longer were significantly more likely to select ‘being a person with a weak character’, ‘having a parent with psychological problems’, and ‘experiencing a traumatic event’ as causing depression (p = 0.004), (p = 0.017) and (p = 0.034) respectively. People who spoke the Filipino were less likely than those who spoke Arabic to select ‘coming from a war torn country’ as a cause (p = 0.038). In terms of risk factors, doctors were significantly more likely than nurses to select ‘being of Christian background’ and ‘those who are not very religious ‘as increasing vulnerability for depression (p = 0.032) and (p = 0.043) respectively. Finally, respondents with more experience were more likely to select ‘people who are poor’ as a risk factor for depression (p = 0.030). Those with more years of working experience were more likely to select ‘people who are poor’ as a risk factor for depression (p = 0.030).

#### Psychosis vignette

With regards to the psychosis vignette, females were more likely to endorse ‘experiencing a traumatic event’ and ‘family problems’ as casual factors for psychosis than males (p = 0.005) and (p = 0.005). The belief that psychosis can be the result of the person having ‘a weak character’ decreased as experience levels increased (p = 0.017). Finally, those who were had resided in UAE for more than 10 years were more likely to endorse ‘evil eye’ as causal factor for psychosis (p = 0.005). Referring to risk factors that increase vulnerability to psychosis, ‘people who are single/on their own’ (p = 0.020) and those ‘born in a war torn country’ (p = 0.020) were more frequently selected by females than males.

### Between vignette comparisons of causal and risk factors

**Fig. 1:**
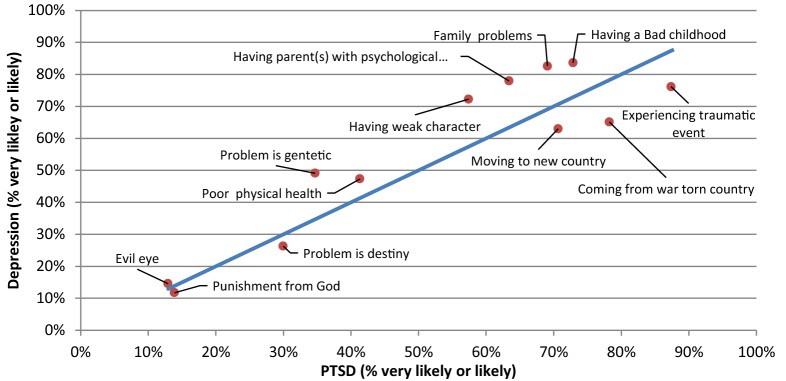
Comparison of the proportion of the likely causal beliefs between the PTSD and depression with suicidal thoughts vignettes

**Fig. 2:**
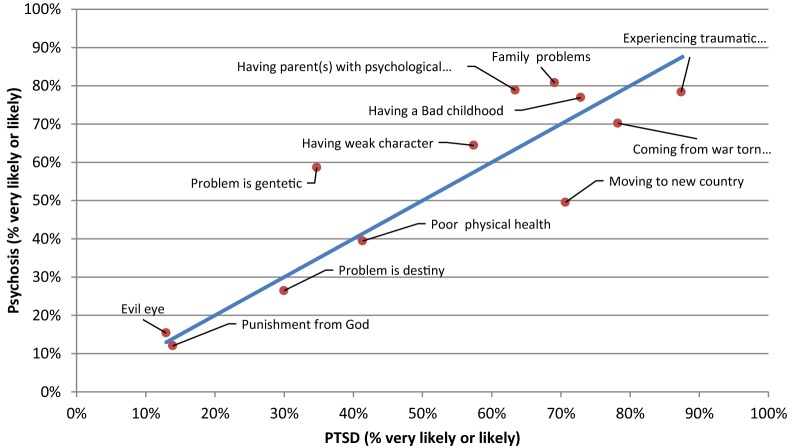
Comparison of the proportion of the likely causal beliefs between the PTSD and psychosis vignettes

**Fig. 3:**
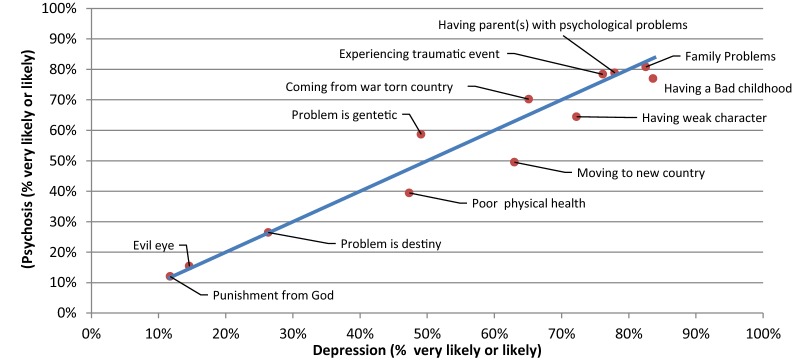
Comparison of the proportion of the likely causal beliefs between the depression with suicidal thoughts and psychosis vignettes

**Fig. 4:**
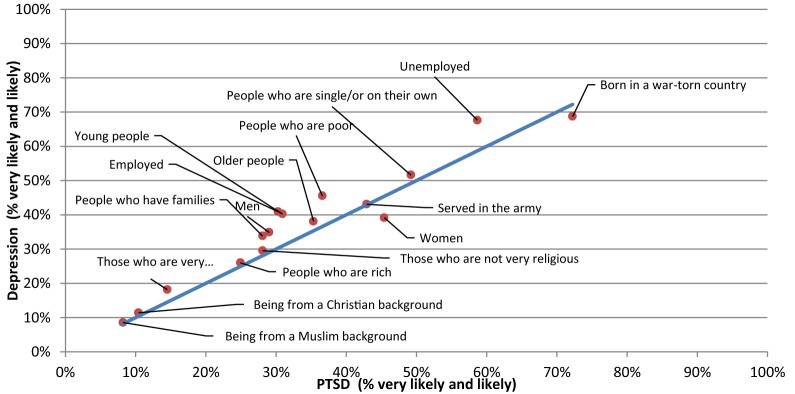
Comparison of the proportion of the likely risk factors between the PTSD and depression with suicidal thoughts vignettes

**Fig. 5:**
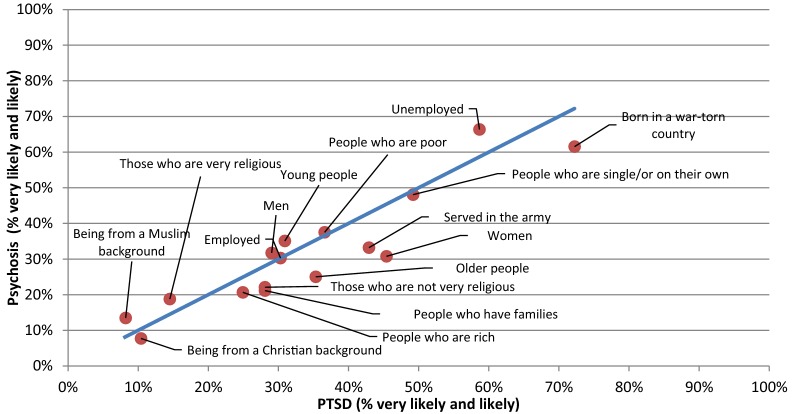
Comparison of the proportion of the likely risk factors between the psychosis and PTSD vignettes

**Fig. 6:**
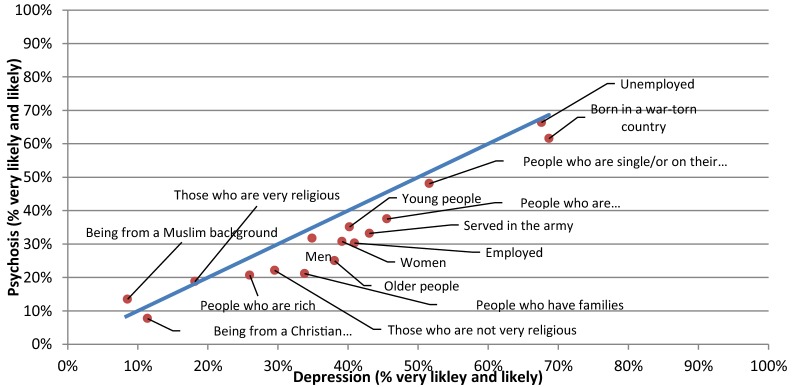
Comparison of the proportion of the likely risk factors between the psychosis and depression with suicidal thoughts vignette

Scatterplots were constructed in order to provide a comparison between any two pairs of vignettes with respect to ‘likely’ causes and risk factors (Figs. 1, 2, 3, 4, 5, 6).

When comparing the responses between vignettes, causes that were chosen significantly more often as ‘likely’ for PTSD than Depression were ‘coming from a war-torn country’ (p = 0.008) and ‘experiencing a traumatic event’ (p = 0.013). Conversely, causes that were chosen significantly more often as ‘likely’ for Depression than PTSD were ‘having a parent or parents with psychological problems’ (p = 0.004), ‘family problems’ (p = 0.005), ‘the problem is genetic or inherited’ (p < 0.001), and ‘being a person with a weak character’ (p = 0.006). ‘Moving to a new country (losing contact with family and friends)’ (p < 0.001) was chosen significantly more often as a ‘likely’ cause for PTSD than for Psychosis, whereas ‘having a parent or parents with psychological problems’ (p = 0.004), and ‘the problem is genetic or inherited’ (p < 0.001), were chosen significantly more often as ‘likely’ causes for psychosis than for PTSD. ‘The problem is genetic or inherited’ (p < 0.001), was chosen significantly more often as ‘likely’ causes for depression than for psychosis. Finally, there were no causes chosen significantly more often as a ‘likely’ cause for Psychosis than for Depression.

The only statistically significant difference noted for risk factors was in the percentage of respondents who saw gender, i.e. ‘Women’ as a risk for PTSD (45.4%) compared to psychosis (30.8%) (p-value = 0.038).

## Discussion

Nurses and doctors are at the forefront of patient care in hospitals. Thus, it is important that their knowledge regarding the causes of and risk factors for mental illnesses is evidence-based and contemporary. As patients in paediatric hospital settings are often chronically ill, early detection and treatment of mental illness is vital in improving the psychological wellbeing of a group that has been identified as having high levels of psychiatric comorbidity [17]. This is the first known study in the UAE to elucidate the knowledge and beliefs of paediatric hospital staff concerning the causes of and risk factors for mental illnesses, namely PTSD, depression with suicidal thoughts and psychosis. The findings highlight areas where further education and training may be required.

Encouragingly, respondents endorsed causal factors such as trauma exposure for PTSD and early childhood adversity (‘having a bad childhood’) for depression which is consistent with contemporary aetiological models for these two mental illnesses [18, 19]. However, the belief that factors such as ‘family problems’ or ‘trauma exposure’ are causal factors for psychosis are somewhat inconsistent with the current biogenetic understanding of this disorder [20]. Of note, is that when the influence of sociodemographic factors on causal selection was undertaken, the beliefs that psychosis can be caused by ‘family problems’ or ‘trauma exposure’ were found to be more frequently selected by females than males, indicating an area for targeted training. Further, the finding that a longer length of residency in UAE was significantly associated with a greater belief that ‘evil eye’ is a cause for psychosis, is of interest and may be related to greater exposure to beliefs related to the role of Ash-Shaytan (Satan) in causing unwanted thoughts and beliefs [13]. This would suggest that health promotion programs should be aware of specific religious beliefs when tailoring programs to such countries. On the other hand, the finding that greater work experience reduced the belief that psychosis is a result of a weakness of character, is useful in highlighting the need for mental health promotion education targeted towards early career health professionals. Beliefs around causes for depression were also found to be influenced by socio-demographic factors. Older residents were more likely to select the option that developing depression ‘is destiny’. This may be reflective of the attribution to external higher powers for the development of mental illness, beliefs that are not uncommon amongst some religious faiths [13, 21]. This finding is consistent with another study of 601 medical practitioners in Pakistan, where it was noted that 37.2% believed that depression was caused by religious or supernatural causes [22], although the role of age was not specifically highlighted. The influence of length of residency in UAE also played a role in selection of causal factors for depression, some which appear to be consistent with aetiological theories (parents with psychological problems) and others not (weakness in character). The finding of stigmatising beliefs is not surprising and has commonly been reported amongst general populations [10] and health professionals [22] located in both western and non-western nations [21]. More specifically, in a systematic review examining stigma associated with mental illness in the Arab culture [21], it was noted that negative stigmatising beliefs were held by different Arab populations across several countries in Middle East and among Arabs living in Western nations [21]. It is also interesting to note that the belief that depression is caused by ‘weakness of character’ was also found to be more significantly endorsed when compared to PTSD. In a national survey of the Australian public, it was noted that a belief in a weak personality as the cause of mental disorders was most consistently associated with specific types of stigma such as personal stigma, perceived stigma and desire for social distance across vignettes [23], suggesting that stigma towards depression may also be held by the health professionals surveyed in this study.

When considering the respondents as a whole, a consistent theme centring on potential trauma exposure (‘coming from war torn country’) and a lack of social resourcing (unemployment and being single) emerged as risk factors for all three vignettes. Given that these factors have been widely recognised as increasing vulnerability to mental health disorders [24, 25] these responses are encouraging. However, when we examined the influence of socio-demographic factors, some interesting patterns related to religion and/or cultural beliefs emerged. More specifically, it was noted that doctors were more likely to believe that ‘people who are not very religious’ or ‘those who were from a Christian background’ were more vulnerable to depression. This vulnerability related to religion or lack thereof it, was also noted when considering the PTSD vignette. Here, more females compared to males significantly believed that ‘people who are not very religious’ had a greater risk for PTSD. The role of religion on mental health outcomes has been investigated extensively with most research indicating an overall inverse relationship between religiosity and mental health, more specifically, depression [26]. However, it has also been noted that while religious beliefs give people hope, a coping mechanism and a sense of community, they may also lead to increased levels of depression if a person feels guilty for not upholding the standards of their religion [26]. The finding that a longer length of stay was more significantly associated with belief in the role of an ‘evil eye’ increasing vulnerability to psychosis is of interest. Such a belief generally lies outside of traditional religious teachings and more likely reflects socio-cultural traditions [13, 27], nonetheless, it remains prevalent and should be considered when developing mental health education and promotion programs. The finding that as levels of clinical experience increased there was a significantly reduced belief that those with a ‘weak character’ were more vulnerable to psychosis is positive and in some ways consistent with findings that increased levels of education are associated with reduced negative beliefs that a mental illness is a weakness of character, findings noted in both western based research [23] and those specific to Arab countries [27].

In sum the findings from the present study as a whole are encouraging but also have important implications for the future training and education of hospital staff, namely in delivering targeted programs designed to improve mental health literacy. While most of the responses of the group overall aligned to known causal and risk factors for the three mental illnesses investigated, the associations between socio-demographic factors identified cohorts where knowledge differed and had potential to lead to greater levels of stigma. There is no doubt about the importance of religion in shaping and influencing the beliefs towards mental illness and help-seeking in Arab populations and other closely aligned communities. As such mental health literacy and promotion campaigns should embrace the positive messages from religious teachings while targeting the misleading associations related to punishment from supernatural powers that remain prevalent in Arab societies. The development of culturally specific and sensitive campaigns requires not only reference to the evidence based literature related to mental illness but a deep seated understanding of the cultural, political and social aspects that define the specific Arab culture. This specificity is further compounded in a country such as UAE, where a large proportion of the Emirati healthcare workforce are expatriates who bring their own culture, religious beliefs and knowledge of mental health with them. As such any further training needs to be contextualized with locally based studies such as this.

The study has a number of limitations that should be noted. First, the study had a response rate potentially as low as 27% and was restricted to public hospitals in the UAE that agreed to participate. This sampling meant that only a limited number of healthcare professionals within a small subset of Emirati hospitals participated in the study. This limits the study’s generalizability to clinicians, and in particular doctors given their smaller proportion in the final sample, across the UAE. These surveys were also long and required the participant to understand English. Whilst most Emirati healthcare professionals are proficient in English, the possibility that some aspects of the survey may have been misinterpreted if proficiency was low cannot be discounted. Moreover, vignettes used adults instead of children. Whilst the scenarios were culturally adapted, future research should tailor the surveys to the primary language spoken as well as for the target population. Finally, as the study is the first to evaluate the knowledge and beliefs of the causes and risk factors for common mental health disorders in this specific population, no direct comparative data was available. Going forward, future studies should include open ended responses to allow for richer descriptions that are not predefined and more vignettes of common mental illnesses such as anxiety disorders, mood disorders and eating disorders to better represent the full spectrum of psychiatric morbidity in the Emirati population.

## Conclusion

The cultural melting pot of the UAE makes it a unique yet challenging place in which to deliver an inclusive and effective healthcare system. This study is the first in the UAE to evaluate the knowledge and beliefs of the causes for and risk factors of common mental illnesses in paediatric hospital staff. We found that whilst nurses and doctors were able to identify some common causes of and risk factors for PTSD, depression with suicidal thoughts and psychosis, responses from certain socio-demographic groups highlighted the presence of potentially stigmatising beliefs. Our findings provide evidence to support the design and targeted delivery of mental health literacy campaigns for current and future healthcare professionals working in a diverse Arab nation such as the UAE.

## Data Availability

The datasets used and/or analysed in the present study are available from author NA on reasonable request.

## Abbreviations

DSM: Diagnostic and Statistical Manual of Mental Disorders
MHL: Mental Health Literacy
NSMHWB: Australian National Survey of Mental Health and Wellbeing
PTSD: Posttraumatic stress disorder
UAE: United Arab Emirates
